# Antioxidative system response of pedunculate oak (*Quercus robur* L.) seedlings to Cd exposure

**DOI:** 10.1007/s12298-019-00712-1

**Published:** 2019-10-04

**Authors:** Magdalena Sozoniuk, Michał Nowak, Karolina Dudziak, Piotr Bulak, Justyna Leśniowska-Nowak, Krzysztof Kowalczyk

**Affiliations:** 1 Institute of Plant Genetics, Breeding and Biotechnology, University of Life Sciences in Lublin, Akademicka 15 St., 20-950 Lublin, Poland; 2 Institute of Agrophysics, Polish Academy of Sciences, Doswiadczalna 4 St., 20-290 Lublin, Poland; 3 Chair and Department of Biochemistry and Molecular Biology, Medical University of Lublin, Chodźki 1 St., 20-093, Lublin, Poland

**Keywords:** Pedunculate oak, Antioxidative enzymes activity, Gene expression, Cd

## Abstract

The use of pedunculate oak (*Quercus robur* L.), along with other tree species, for the afforestation of heavy metal contaminated lands is an attractive prospect. Little, however, is known of *Q. robur* tolerance and its antioxidative system response to heavy metal exposure. The main objective of the study was to determine the cadmium-induced changes in antioxidative system of pedunculate oak in an attempt to identify molecular mechanisms underlying Cd tolerance. This may be of great importance in respect of using *Q. robur* for phytoremediation purposes. As the response of the antioxidative system to heavy metal contamination can vary within species, the research was conducted on oak seedlings from two different regions of origin. Differences in antioxidative system response of seedlings derived from tested regions of origin were noticed both at the transcript and enzyme activity levels. The obtained results indicate that ascorbate peroxidase (APX; EC 1.11.1.11) and superoxide dismutase (SOD; EC 1.15.1.1) play a first barrier role in oak seedlings response to the oxidative stress caused by Cd exposure. Catalase (CAT; EC 1.11.1.6) is involved in reducing the negative effects of prolonged Cd treatment.

## Introduction

Heavy metal contamination of soils is a serious problem of the modern world (Mihucz et al. 2012). Cadmium is recognized as one of the most toxic pollutant due to its high mobility and low amount needed to cause the toxicity symptoms. Cd accumulation in plants affects many cellular and physiological processes such as nitrogen metabolism or mineral uptake (Benavides et al. 2005; DalCorso et al. 2008; Wu et al. 2015). Cd was shown to induce ultrastructural damage to chloroplasts, which subsequently affects photosynthesis and causes decline in biomass production. Moreover, decrease in Rubisco protein content was reported in Cd affected plants (Arena et al. 2017; Figlioli et al. 2019). However, plants showing high Cd tolerance revealed increased Rubisco level, thus mitigating Cd effect on biomass production. Other mechanisms allowing species to endure Cd pollution include metal complexation by chelating agents or its compartmentalization (Sorrentino et al. 2018). As cadmium causes oxidative stress (Mihucz et al. 2012), an adequate antioxidative system reaction is also a fundamental cell defense mechanism. The activation of antioxidative enzymes helps to protect plant against oxidative injury evoked by heavy metals (DalCorso et al. 2008). Among others, major antioxidative enzymes are superoxide dismutase (SOD; EC 1.15.1.1), catalase (CAT; EC 1.11.1.6) and ascorbate peroxidase (APX; EC 1.11.1.11) (Mittler 2002). SOD catalyzes the dismutation of O_2_^·−^ to H_2_O_2_, which is subsequently detoxified by CAT and APX (Sharma and Dietz 2009).

There are numerous studies reporting heavy metal-induced changes in antioxidative system of crop plants (Ekmekçi et al. 2008; El-Beltagi et al. 2010; Khan et al. 2007; Malecka et al. 2014; Rodriguez-Serrano et al. 2009). The studies conducted on forest trees response to heavy metal stress focus mainly on various *Populus* and *Salix* species, mostly due to their industrial importance and fast growth rate (Gaudet et al. 2011; Ge et al. 2012; He et al. 2013; Landberg and Greger 2002; Nikolić et al. 2008). Little, however, is known about other tree species response to heavy metal stress (Schützendübel et al. 2001). Some of defense mechanisms against Pb and Cd induced stress have been investigated in *Quercus ilex* (Arena et al. 2017).

Studies performed on *Quercus pubescens* and *Quercus suber* suggest their possible use in phytoremediation practices (Paoletti and Günthardt-Goerg 2006; Gogorcena et al. 2011). Thus, the understanding of molecular mechanisms underlying heavy metal tolerance in *Quercus* species could be vital for selection and breeding of trees suitable for the phytoremediation. Efficient detoxification strategies, such as prompt activation of antioxidative enzymes, may alleviate negative effects of Cd on trees growth and development. This may be of great importance in context of utilizing *Quercus robur* for the afforestation of heavy metal contaminated lands. The main objective of present research was to determine the antioxidative system response of pedunculate oak to the Cd treatment in an attempt to identify molecular mechanisms underlying Cd tolerance. As the response of the antioxidative system to heavy metal contamination can vary within species (Anjum et al. 2008; Gonnelli et al. 2001; Landberg and Greger 2002; Wu et al. 2003), the research was conducted on oak seedlings from two different regions of origin. To our best knowledge, this is the first study that aimed to assess changes induced by Cd in *Q. robur* leaves both at transcript and enzyme activity levels.

## Materials and methods

### Plant material and growth conditions

One-year old pedunculate oak (*Quercus robur* L.) seedlings were obtained from two different regions of origin—No. 659 (Swidnik Forest District, Poland) and No. 455 (Lubartow Forest District, Poland). Plants were grown hydroponically in the modified Hoagland solution which contained (mM) 1.25 Ca(NO_3_)_2_·4H_2_O, 1.25 KNO_3_, 0.25 NH_4_H_2_PO_4_, 0.5 MgSO_4_ and (μM) 22.5 FeSO_4_·7H_2_O, 11.6 H_3_BO_3_, 0.19 ZnSO_4_·7H_2_O, 0.08 CuSO_4_·5H_2_O, 2.29 MnSO_4_·4H_2_O, 0.03 Na_2_MoO_4_·2H_2_O. Plants were kept in PP tubs (30 seedlings per tub, each tub containing 30 l of nutrient solution). The nutrient solution was constantly aerated and changed every 4 days. The pH of nutrient solution was adjusted to 5.8 every 2 days. Plants were grown in 14 h day (120 µmol m^−2^ s^−1^)/10 h night photoperiod at 25 ± 3 °C. After 21 days of acclimatization, the nutrient solution was supplemented with CdCl_2_ (10 or 50 µM) or remained unsupplemented (control plants). The leaf samples for transcriptomic and biochemical analyses were cut from fully expanded leaves [10th stage of leaf development according to Spieß et al. (2012)] after 1 h, 3 h, 24 h, 3 days and 7 days of Cd stress.

### Gene expression analysis

Total RNA was extracted from leaves according to the method of Le Provost et al. (2007) based on CTAB extraction and LiCl precipitation. The concentration and purity of RNA was assessed with NanoDrop 2000 spectrophotometer (Thermo Scientific). The integrity of RNA samples was analysed by means of electrophoresis in 2% agarose gel stained with ethidium bromide. Genomic DNA was removed by DNase I (Ambion) treatment. The reaction of reverse transcription was performed on 1 µg RNA with SuperScript VILO cDNA Synthesis Kit (Life Technologies) according to the supplier’s recommendations. Obtained cDNA was used as template in the qPCR analysis.

The transcript levels of *Cu/Zn*-*SOD* (GenBank Accession No. FN719644), *CAT* (GenBank Accession No. FN999264) and *APX* (GenBank Accession No. FP062995) were determined by real-time PCR analysis. Reference gene selection was performed. The *GAPDH* gene was used as an internal control to normalize the data. The sequence of *GAPDH* (ID6744996) was obtained from the *Fagaceae* database, Fagaceae Genome Web (http://www.fagaceae.org/). Primers and TaqMan probes used in experiment (Table 1) were designed with AlleleID^®^ (Premier Biosoft) and Primer3Plus. Primers for *GAPDH* were taken from Marum et al. (2012). Probes were labelled with FAM/BHQ1. Real time-PCR was carried out according to the following cycling program: 2 min at 50 °C, 10 min at 95 °C, 40 cycles of 15 s at 95 °C and 1 min at 60 °C in a total reaction volume of 20 µl containing 1 × TaqMan Gene Expressiom Master Mix (Applied Biosystems), 400 nM of each primer, 250 nM of probe and 100 ng cDNA. Transcriptomic analyses were conducted in three biological replicates (each pooled from ten plants) with three technical replicates for each sample. No-template controls were included in analyses. The amplification was carried out on Mx3005P apparatus with MxPro software (Stratagene). The relative quantification of transcript levels was performed according to Livak and Schmittgen (2001).

**Table 1:** Sequences of primers and probes used for real-time PCR

Gene	Oligo name	Sequence (5′ → 3′)
Cu/Zn-SOD	SOD-F	CACTGGTACAGTCAAACC
	SOD-R	GTGGACATGAACTAAGTCTG
	SOD-probe	CCACAAGCCAATCTTCCACCT
CAT	CAT-F	GTGTGAAGACTTTCTGGA
	CAT-R	GTCCGTGATGGTATGAAA
	CAT-probe	AGTCTACAATCCTCCAATTCTCCTGA
APX	APX-F	CTAGACACCAGACAGCAGCC
	APX-R	AGGATGCCTTCTTTGCCGAT
	APX-probe	CGGCAAATCCAAGTTCTGAGAGC
GAPDH	GAPDH-F	ACCGACTTCATTGGTGACAG
	GAPDH-R	AGATGCGATGTGGACAATCA
	GAPDH-probe	TACAGTTCCCGTGTGGTTGA

### Crude homogenate and enzyme extract preparation

The 200 mg of leaves tissue was ground to a fine powder in liquid nitrogen and homogenized in 4.5 ml of pre-cooled 50 mM phosphate buffer (pH 7.5) containing 0.1 mM EDTA and 2% PVP. The homogenate was filtered through a nylon cloth. A part of the filtrate, defined as crude homogenate, was used to assess the level of lipid peroxidation (LPO). The second part of filtrate was centrifuged at 11,000 rpm for 20 min at 4 °C (Balakhnina et al. 2015). The supernatant was used to determine the protein content and enzymes activities. Data shown are mean ± SD of six biological samples, each taken pooling material from five plants.

### Lipid peroxidation (LPO) analysis

The level of lipid peroxidation, in terms of thiobarbituric acid reactive substances (TBARS), was estimated according to the method developed by Uchiyama and Mihara (1978), using the extinction coefficient of 156 mM^−1^ l cm^−1^. The TBARS concentration was expressed as nmol TBARS g^−1^ fresh weight (FW). Lipid peroxidation was determined using Hach Lange DR 2800 spectrophotometer.

### Determination of antioxidative enzymes

The activity of SOD (EC 1.15.1.1) was determined according to the method of Giannopolitis and Ries (1977). One unit (1 U) of SOD activity was defined as the amount of enzyme required to cause a 50% inhibition in the rate of NBT reduction. The activity of APX (EC 1.11.1.11) was determined according to Nakano and Asada (1981) and expressed as µmol of substrate (ascorbate) min^−1^ mg^−1^ protein. The activity of CAT (EC 1.11.1.6) was estimated following the method of Aebi (1984), using the extinction coefficient of 39.4 mM^−1^ l cm^−1^. The CAT activity was expressed as µmol of substrate (H_2_O_2_) min^−1^ mg^−1^ protein. Total soluble protein content was determined according to the method of Bradford (1976) using the bovine serum albumin (BSA) as a calibration standard. Protein content and SOD activity were measured using Hach Lange DR 2800 spectrophotometer, while CAT and APX activities were assessed using Shimadzu UV–Vis 160A spectrophotometer.

### Statistical analysis

To detect significant differences in the expression levels of *Cu/Zn*-*SOD*, *CAT* and *APX* between Cd-treated and control plants, the nonparametric Wilcoxon signed-rank test was used. For TBARS content, SOD, APX and CAT activities, two-way ANOVAs with repeated measures were applied (Cd concentration and region of origin as two factors) followed by post hoc Bonferroni corrections. When the condition of sphericity has not been met (Mauchly’s test), Greenhouse–Geisser correction was applied. All statistical tests were performed with SPSS 22.0 software.

## Results

### Analysis of gene expression

**Fig. 1:**
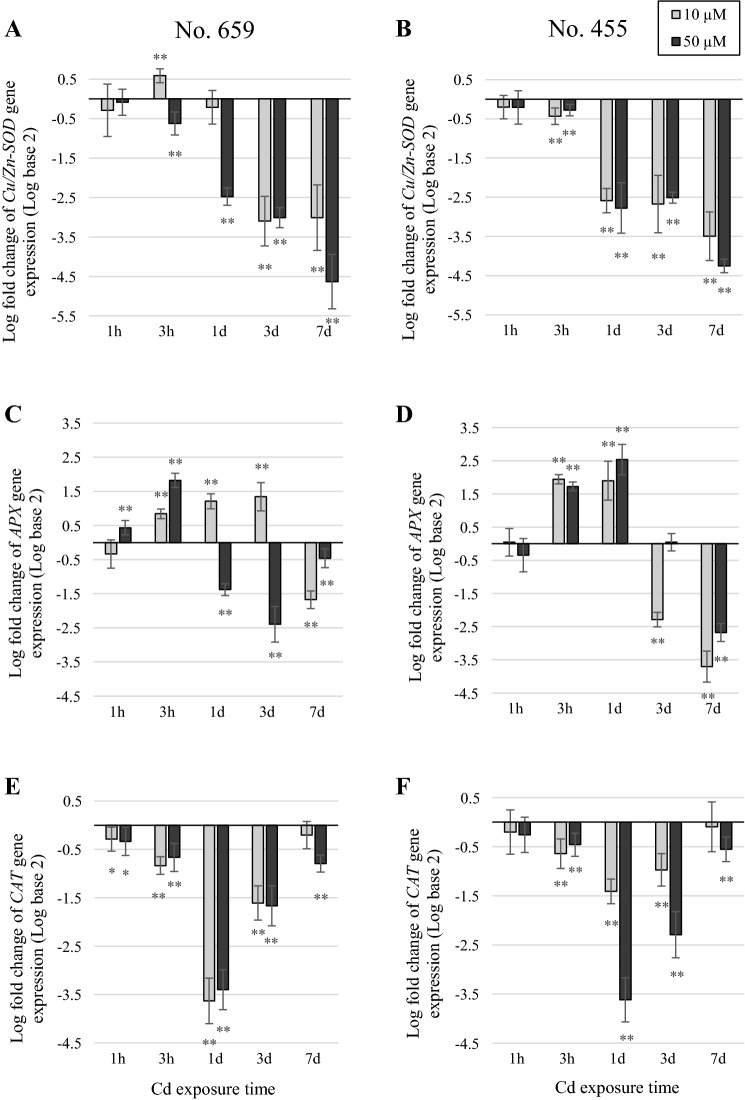
Gene expression in leaves of Nos. 659 and 455 seedlings exposed to 10 µM CdCl_2_ or 50 µM CdCl_2_. Changes in transcript levels were presented as a fold change in comparison to their respective controls. Data are the means of three replicates (± SD). Asterisks indicate significant differences between Cd treated and control plants in the same exposure time (Wilcoxon signed-rank test, *p ≤ 0.05; **p ≤ 0.01; ***p ≤ 0.001)

The Cd treatment resulted in a sustained decrease in the *Cu/Zn*-*SOD* expression compared with the control (Fig. 1a, b), except for an increase (*p *≤ 0.01) found at 3 h after the application of 10 µM Cd in No. 659 seedlings. The expression of *APX* was upregulated in first hours of stress and then decreased, as compared to respective controls (Fig. 1c, d). The seedlings from tested regions of origin differed in their reaction time. In low dose treated plants, *APX* was downregulated after 3 days (*p *≤ 0.01) or 7 days (*p *≤ 0.01) of exposure for No. 455 and No. 659 regions of origin, respectively. No. 659 plants showed a decrease (*p *≤ 0.01) in the *APX* expression level 1 day after the application of 50 µM Cd while in No. 455 plants, a decrease (*p *≤ 0.01) was found after 7 days of treatment. The transcript level of *CAT* was downregulated by Cd treatment with great decrease (*p *≤ 0.01) found after 1 day of exposure, compared to respective controls. Both Cd doses caused similar changes in *CAT* expression in No. 659 plants (Fig. 1e) while No. 455 plants were more affected by higher Cd dose (Fig. 1f).

### Analysis of lipid peroxidation (LPO)

**Fig. 2:**
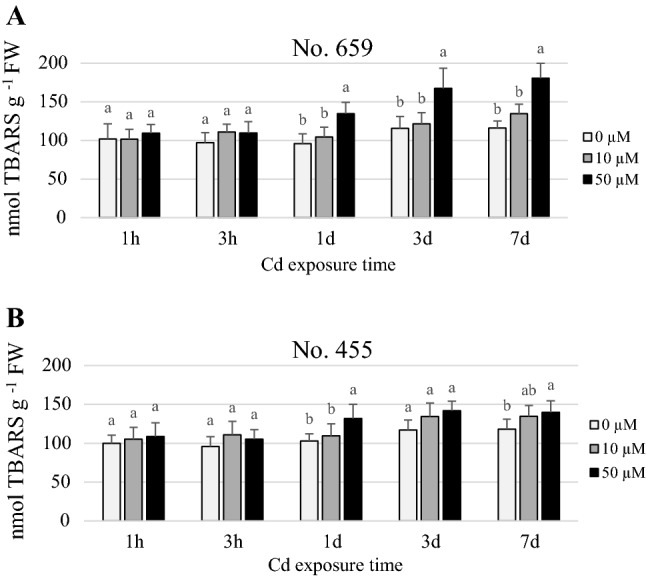
TBARS content in leaves of *Q. robur* seedlings from No. 659 (**a**) and No. 455 (**b**) regions of origin, exposed to 0 µM, 10 µM or 50 µM Cd. Data are the means of six replicates (± SD). Different letters on the bars indicate significant difference between treatments within the same exposure time (Bonferroni test, *p *≤ 0.05)

High Cd dose caused increase (*p *≤ 0.05) in TBARS content in the leaves of No. 659 plants after 1 day, 3 days and 7 days of exposure, while in No. 455 plants, after 1 day and 7 days (*p *≤ 0.05) of exposure, compared to their respective controls (Fig. 2a, b). The maximal content of TBARS was observed at the end of treatment and averaged 180.7 ± 19.4 nmol TBARS g^−1^ FW for No. 659 and 139.9 ± 14.6 nmol TBARS g^−1^ FW for No. 455. There was no difference (*p *> 0.05) in TBARS concentration between the control and the low dose plants. Interestingly, differences in LPO between plants of different region of origin treated with 50 µM Cd were revealed. After 3 days and 7 days of exposure, the TBARS content in No. 659 was 18% (*p *≤ 0.05) and 29% (*p *≤ 0.001) higher than in No. 455, respectively.

### Analysis of antioxidative enzyme activity

**Fig. 3:**
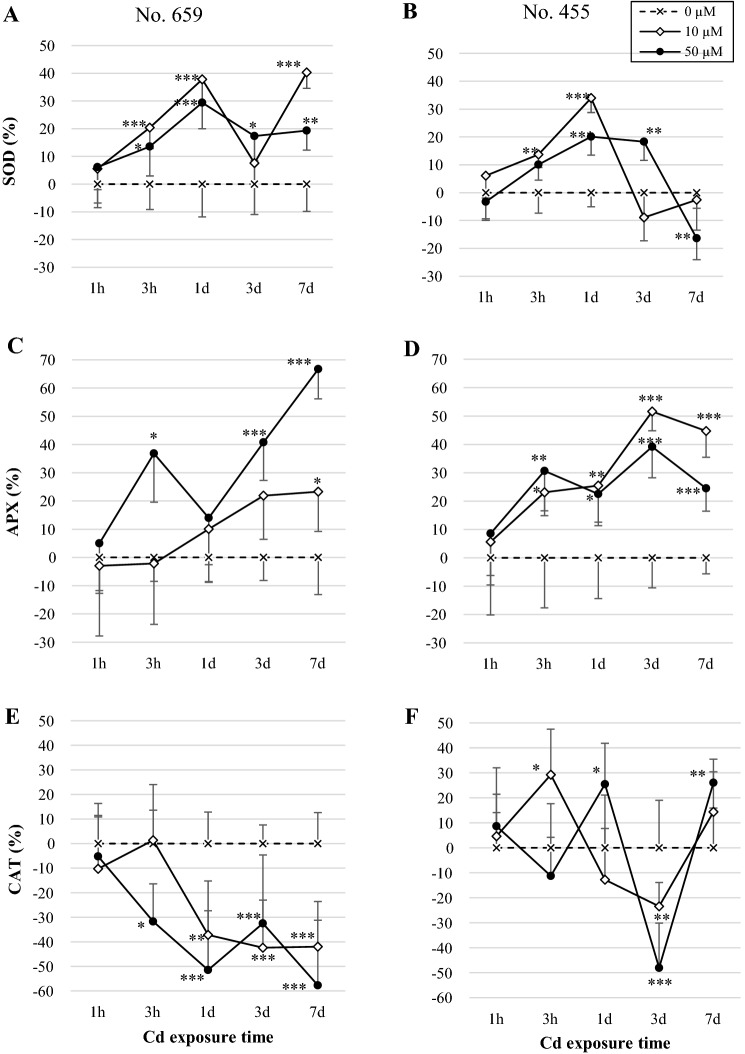
Change of antioxidative enzyme activity in leaves of Nos. 659 and 455 seedlings exposed to 10 µM CdCl_2_ or 50 µM CdCl_2_. Enzyme activities were expressed relative to the activity in control plants (= 0%, dashed line). Data are the means of six replicates (± SD). Asterisks indicate significant differences between Cd treated and control plants in the same exposure time (Bonferroni test, *p ≤ 0.05; **p ≤ 0.01; ***p ≤ 0.001)

SOD activity was initially stimulated by Cd exposure in plantlets from both regions of origin (Fig. 3a, b). However, after 7 days of Cd treatment, SOD activity in No. 455 leaves declined to the level observed in control (10 μM Cd) or decreased by 16% as compared to control (*p *≤ 0.01; 50 μM Cd). In contrast, No. 659 leaves in 7 days of experiment showed increase in SOD activity by 40% (*p *≤ 0.001; 10 μM Cd) or 19% (*p *≤ 0.01; 50 μM Cd) as compared to their respective controls.

As shown in Fig. 3c, d, APX activity in plants treated with Cd was generally increased. However, No. 659 seedlings responded differently to tested Cd doses. In low dose treated plants, no changes (*p* > 0.05) in APX activity were observed until 7 days of exposure (increase by 23% relative to control; *p *≤ 0.05). High Cd dose caused increase in APX activity already after 3 h of treatment (by 37%; *p *≤ 0.05). Plants from No. 455 region of origin reacted similarly to both Cd concentrations, showing constant stimulation of APX activity from 3 h of treatment to the end of experiment.

In contrast to APX, CAT activity in No. 659 plants decreased by 32% after 3 h in 50 µM Cd treated plants (*p *≤ 0.05) or by 37% after 1 day in 10 µM Cd treated plants (*p *≤ 0.01) as compared to their respective controls. It remained declined till the end of experiment (Fig. 3e). CAT activity in No. 455 seedlings was initially stimulated (by 29% after 3 h of 10 μM Cd treatment; *p *≤ 0.05 or by 25% after 1 day of 50 μM treatment; *p *≤ 0.05), then declined by 23% (*p *≤ 0.01) or by 48% (*p *≤ 0.001) after 3 days of 10 or 50 μM Cd treatment, respectively. Subsequently, an increase of CAT activity was observed, reaching control level (*p* > 0.05) in low dose plants or surpassing control level by 26% (*p *≤ 0.01) in high dose plants (Fig. 3f).

It is notable that in most cases significant differences (*p *≤ 0.01) in enzyme activities were observed between the two regions of origin under the same Cd treatments (data not shown). Enzyme activities were generally higher in No. 455 plants than No. 659 plants, both in control conditions and upon Cd treatment. Only CAT activities in control plants did not differ (*p* > 0.05) between both regions of origin.

## Discussion

Cd accumulation in plants induces oxidative stress (DalCorso et al. 2008). Oxidative damage caused by ROS (reactive oxygen species) may be expressed as the level of lipid peroxidation (Landberg and Greger 2002). In present study, LPO increase was observed in 50 µM Cd treated plants already after 1 day of exposure, whereas 10 µM Cd treatment caused no changes in LPO level throughout the whole experiment. The LPO level was dose- and time- dependent which is in accordance with the results obtained by Martínez Domínguez et al. (2010) and Mishra et al. (2006).

Cd can stimulate or inhibit the activity of major antioxidative enzymes, such as SOD, CAT and APX (Wójcik et al. 2006). Present study showed that Cd exposure increased SOD and APX activity in pedunculate oak leaves, while CAT displayed differential trend of response. The stimulation of SOD observed already in 3 h of treatment (Fig. 3a, b) suggests that this enzyme is a component of early phase response mechanism to Cd exposure in pedunculate oak leaves. As SOD is a source of H_2_O_2_ in cells (Apel and Hirt 2004; Rubio et al. 2004), its stimulation inevitably leads to an increase in cellular H_2_O_2_ concentration. H_2_O_2_ at low concentration acts as signal molecule and regulates peroxidases expression (Schützendübel et al. 2001). The expression analyses of APX showed fluctuation in transcript levels (Fig. 1a, b). Induction of APX transcription in early hours of stress corresponded with increase in APX activity. It is noteworthy that APX was the only antioxidative enzyme gene induced shortly after Cd exposure in *Q. robur* plants from both regions of origin. Thus, we presume that APX, along with SOD, constitutes first barrier to oxidative stress caused by Cd in pedunculate oak leaves.

Heavy metal tolerance in plants requires fast activation of appropriate protective mechanisms, therefore regulation of antioxidative enzymes gene expression is of special interest, as it may strongly affect plant stress response (DalCorso et al. 2008). Our study revealed that Cd induced stress caused significant changes in transcription of selected genes encoding antioxidative enzymes. For instance, CAT expression was significantly decreased at transcriptional level in plants from both regions of origin (Fig. 3e, f). In accordance to this, No. 659 seedlings showed decreased CAT activity (Fig. 3e). Similar reaction of CAT activity to Cd stress was reported by Mishra et al. (2006), Shi et al. (2010) and Mohapatra and Dey (2012). The decline of CAT activity in response to Cd stress might be not only due to decrease in its transcription, but it may also be the result of CAT inhibition by O_2_^·−^ and HO^·^ (Skórzyńska-Polit et al. 2003). Contrary to No. 659 seedlings, No. 455 seedlings exhibited different trend of response (Fig. 3f), showing CAT stimulation in early hours of stress, then the decline in its activity followed by final increase. The induction in CAT activity in response to Cd stress was previously reported by Dinakar et al. (2009), Hsu and Kao (2004) and Nikolić et al. (2008).

Of particular note is the profile of antioxidative enzyme activities obtained in 7th day of stress in plants subjected to higher Cd dose (Fig. 3a–f). In No. 455 seedlings, SOD activity was declined, APX remained elevated whereas CAT activity was increased. In No. 659 seedlings under the same conditions, both SOD and APX activity remained elevated while CAT activity was declined. Plants from both regions of origin experienced oxidative stress in this time point as was indicated by LPO level, however, the oxidative damage observed in No. 659 plants was higher (data not shown; *p *≤ 0.001) than that observed in No. 455 plants. As SOD catalyzes the dismutation of O_2_^·−^ to H_2_O_2_, and both CAT and APX participate in H_2_O_2_ detoxification, the balance between the activity of SOD and H_2_O_2_-scavenging enzymes is crucial during oxidative stress (Apel and Hirt 2004; Mittler 2002). According to Mittler (2002), APX might participate in the removal of fine amounts of H_2_O_2_ whereas CAT is involved in detoxification of high levels of H_2_O_2_ produced during stress. The obtained results might be explained by possible accumulation of H_2_O_2_ in No. 659 plants caused by increased SOD activity combined with decreased CAT activity. Although the No. 659 plants sustained elevated APX activity throughout the experiment, the lack of CAT activation might have been a reason for insufficient H_2_O_2_ removal and subsequent oxidative damage. This would imply that CAT is the key enzyme participating in long-term response to oxidative stress caused by Cd in *Q. robur* leaves. The results are in line with the study performed by Wen et al. (2012) which revealed that Cd exposure induced two waves of H_2_O_2_ accumulation in tobacco cells. In early phase of Cd stress, low concentration of H_2_O_2_ trigged adaptive responses, while prolonged Cd stress caused high accumulation of H_2_O_2_ that induced severe oxidative damage leading to cell death.

Heavy metal tolerance is often linked to high constitutive level of antioxidative enzymes (Landberg and Greger 2002; Sharma and Dietz 2009). In our study No. 455 plants, which exhibited significantly higher SOD and APX activities in control conditions, experienced lower oxidative damage by Cd exposure than No. 659 plants. We suppose that due to high constitutive level of antioxidative enzyme activities, No. 455 seedlings may have ability to react faster to potential oxidative stress than seedlings from the other region of origin.

In conclusion, obtained results indicate that APX and SOD play a first barrier role in oak seedlings response to the oxidative stress caused by Cd treatment. CAT is involved in reducing the negative effects of prolonged exposure of oak seedlings to Cd. To our best knowledge, this is the first report on *Q. robur* antioxidative system response to Cd treatment, including both transcript and enzyme activity analyses. It provides better insight into the basics of pedunculate oak’s tolerance to Cd exposure, which is of great significance in the view of possible use of this tree species for afforestation of heavy metal contaminated sites.
